# Extraction of a Large Drain Included for Nine Months in the Chest

**Published:** 1918-12

**Authors:** 


***Extraction of a Large Drain Included for Nine Months in the Chest.*** By Dr. PETIT DE LA VILLÉON. *Bulletin de la Société de Chirurgie de Paris*, June 25, 1918.

The following case was dealt with by Dr. Petit de la Villéon :

A man, aged 43, suffered first from typhoid fever, then from pneumonia, and lastly from purulent pleurisy; this was treated by means of thoracotomy and drainages. Three weeks after the operation, a drain 10 cm. long and as wide as the ring-finger disappeared in the chest; no one noticed this. The wound closed after suppurating for some weeks. Soon after, the patient began spitting blood and pus. X-ray detected a large drain enclosed in the right hemothorax.

Dr. Petit operated the patient under local anesthesia. He practised a thoracotomy and opened the pleura. No blood, no pus, no serosity even, flowed out of the wound. The hand being introduced into the pleural cavity loosened some adhesions existing there, but found no drain at all. Only by pressing firmly on the posterior part of the lung could the drain's extremity be felt. The whole drain was pulled out by means of forceps. It contained a certain amount of pus; the pulmonary wound bled rather severely; after some swabbing the wall of the chest was partly closed.

The patient's condition was rather bad for a week or two, but after this he healed perfectly well.

Such a case of a drain being included for nine months in a closed chest, and involving nothing else but blood spitting, happens to be very rare.

Dr. Petit considers that the drain was probably enclosed in some interlobar fissure, although X-ray showed the drain somewhat too vertical to correspond to such a position. It may be, therefore, that the drain, having been simply aspirated into the pleural cavity, was enclosed there within some pleural adhesions, and " dug out " a sort of hollow into the lung; the presence of that septic foreign body having determined an abscess of the lung, the spitting of blood and pus, from which the patient had suffered, was explained.

Notice should be taken of the fact that drains do not always prove opaque to X-rays, hence being sometimes invisible in radiographs.

Red colored drains are always visible on X-ray photographs, since their color is due to some substance containing certain lead salts.

On the contrary, white and black colored drains are generally transparent to X-rays.

